# Supplementary material for: Self-Induced Buckling in Hollow Microgels
Source: ACS Nano. 2026 Jan 15;20(4):3541–50. doi: 10.1021/acsnano.5c15990 (PMC12874633; doi:10.1021/acsnano.5c15990)
Supplement: Supplementary file 1 [file nn5c15990_si_001.pdf]

Supporting Information:
Self-induced buckling in hollow microgels

Leah Rank and Emanuela Zaccarelli*

CNR Institute of Complex Systems, Uos Sapienza, and Department of Physics, Sapienza University of Rome, Piazzale Aldo Moro 2, 00185 Roma, Italy

E-mail: leah.rank@uniroma1.it;emanuela.zaccarelli@cnr.it

S1: Gyration radius $c = 5\,\%$ of non-hollow microgels

Fig. S1 reports $p(R_\mathrm{g})$ for $c = 5\,\%$ standard microgels, with $N_\mathrm{m} \sim 5000$, calculated from the simulations reported in Ref.,[1] in order to favor the comparison of its behaviour with the corresponding hollow microgels reported in the main text in Fig. 2.

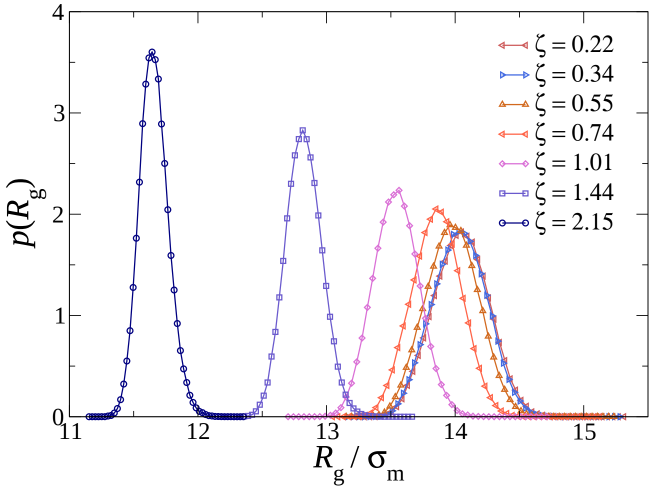

Figure S1: Distribution of gyration radius $p(R_\mathrm{g})$ for non-hollow microgels with $c = 5\,\%$ for different packing fractions from Ref.[1]

S2: Mean-squared displacements of hollow microgels

We briefly mention in the paper that the dynamics of the microgels at high packing fractions (where buckling occurs) are dynamically arrested. This is shown in Fig. S2 where the Mean-squared displacement (MSD) of microgel centers of mass is reported as a function of time at selected packing fractions for both $c = 5\,\%$ and $c = 10\,\%$ microgels. It is clear that for $\zeta \sim 1.0$ and above, the dynamics are almost arrested within the simulation timescale.

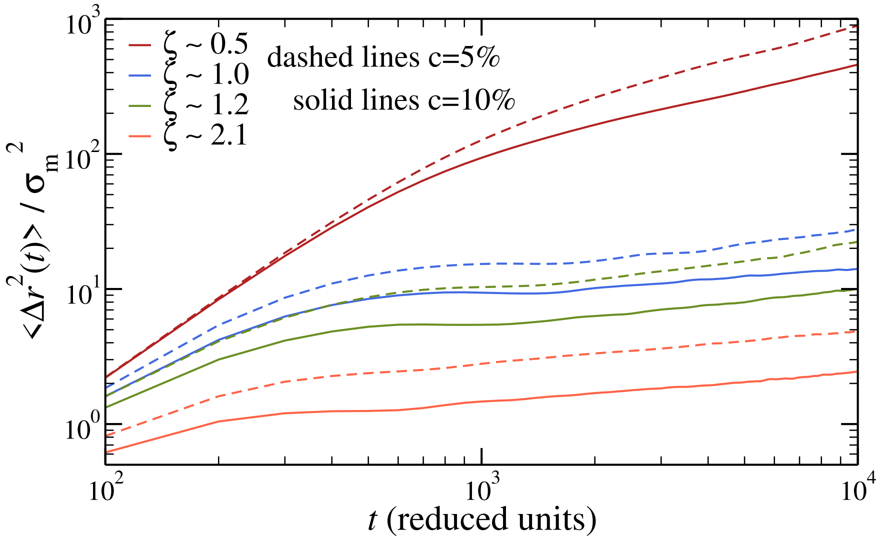

Figure S2: Mean-squared displacement (MSD) of the $c = 5\,\%$ (dashed lines) and $c = 10\,\%$ (solid) suspension at different packing fractions ζ: The curves of the same colour are measured from simulations of approximately the same packing fraction (indicated by the legend $\zeta \sim$) to compare the two crosslinker regimes.

S3: Preparation of the simulated state points at high ζ

To compress the system, we perform an annealing procedure. To this aim, we gradually shrink the box in small steps, allowing sufficient equilibration time in between. After each step, we perform energy minimization followed by a short equilibration to allow the system to relax. Once the desired box size is reached, the system undergoes a long equilibration run of at least 10^7 MD timesteps. To start preparing the next packing fraction, we then use the last configuration and perform the shrinking process again in an iterative fashion.

However, we know that at very high ζ, the system gets slower and slower, so we test the influence of the preparation protocol on the reported results. We thus analyse a quenching protocol, where we shrink the box from $\zeta = 0.64$, where the system is far from any deformation, directly to a high packing fraction. In Fig. S3, we compare the distribution of the radius of gyration (a) and the radial distribution function (b) for annealed and quenched states at two of the largest studied ζ for $c = 10\,\%$ hollow microgels.

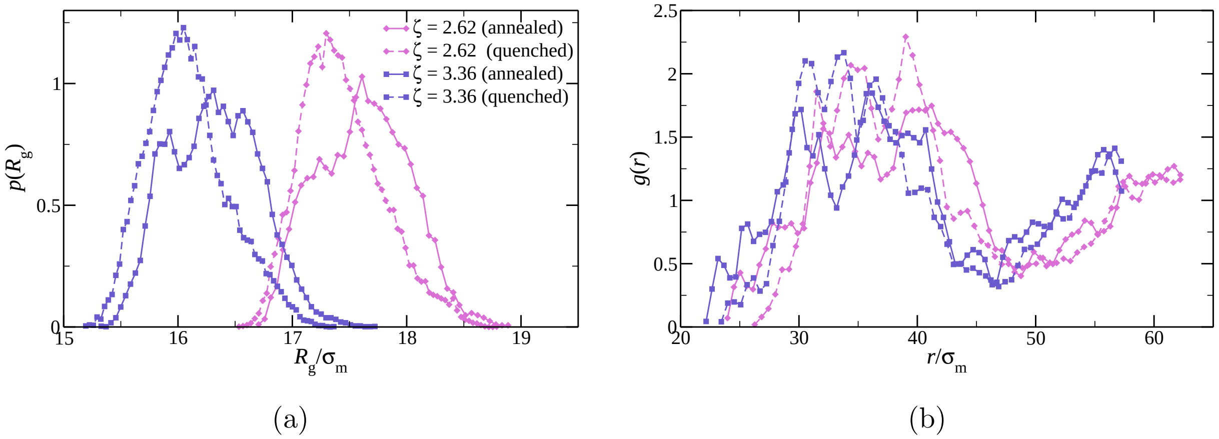

Figure S3: Comparison between annealed and quenched configurations of $c = 10\,\%$ hollow microgels at two representative values of ζ in the high density range: (a) $p(R_\mathrm{g})$ and (b) $g(r)$.

We find that also the quenched runs do not show a completely Gaussian R_g distribution, characterised by a large tail at high R_g. However, the deviation from a bell-shaped distribution is much less pronounced than for annealed states, not developing a two-peak distribution of $p(R_\mathrm{g})$ as observed for the latter. Thus, as expected, quenching causes the microgels to shrink more rapidly to be able to fit in the smaller box, so that they have less time to deform. Compressing the box even further, this tendency is maintained. However, it is important to stress that the multi-peak feature of the $g(r)$ discussed in the main text is also visible for quenched systems. Hence, this is a genuine feature that should be observable in experiments.

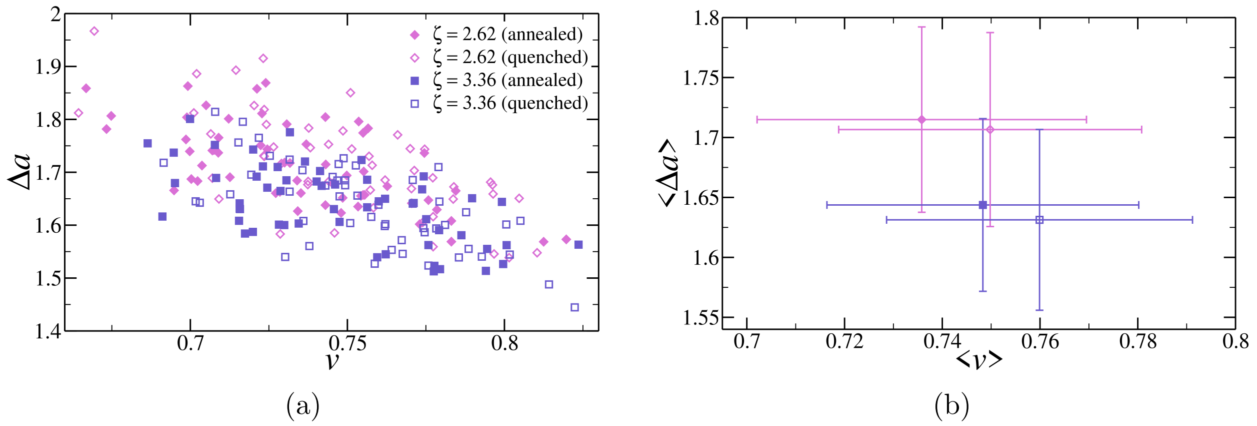

Figure S4: Quenching is slightly visible in the shape phase diagram. In the plot on the left, each point represents an individual microgel at a given timestep, while the plot on the right shows the averaged phase diagram of both the microgel ensembles as well as 5 uncorrelated timesteps. The quenched versions are found more toward the right of the phase diagram, indicating more round and less deformed shapes.

Next, we examine the effect of the preparation protocol on the $c = 10\,\%$ shape phase diagram, reported in Fig. S4 for individual microgels (a) and averaged over all microgels (b). In this case, we just observe a small shift in the data, which does not affect the main findings discussed in the main text.

S5: Average density profiles for $c = 10\,\%$ hollow microgels

In Fig. S5 we show the density profiles of $c = 10\,\%$ hollow microgels at selected packing fractions. The microgels are clearly hollow up to $\zeta \sim 1.0$, above which the cavity starts to fill. For $\zeta \gtrsim 1.5$, the microgels become completely filled. At these high ζ values, despite the individual deformation, the average profiles, still computed radially as a function of the distance from the center of mass, look similar to those of standard microgels.

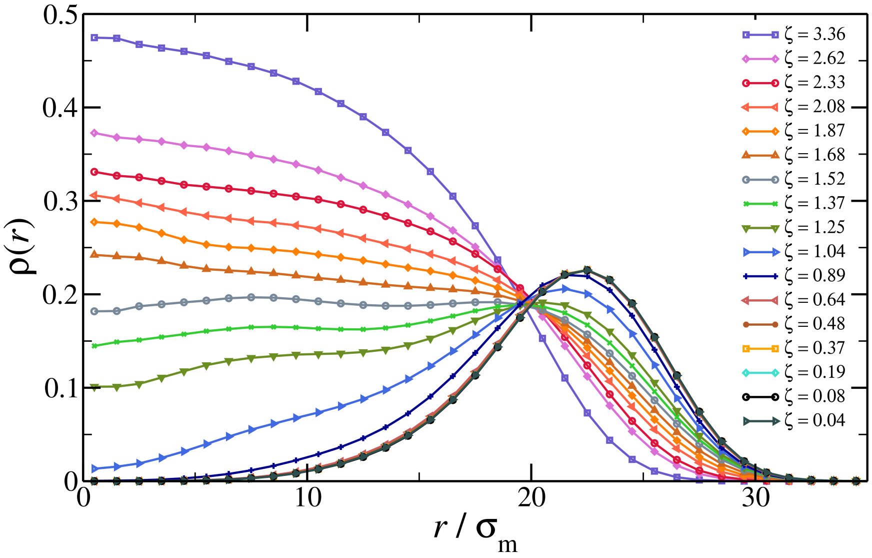

Figure S5: Averaged density profiles of microgels with $c = 10\,\%$ crosslinkers at different packing fractions.

S6: Additional results for hollow microgels with thinner shells, $\delta_{\mathrm{rel}} = 0.21$, and $c = 10\,\%$

We also performed extensive numerical simulations of microgels with thinner shells, $\delta_{\mathrm{rel}} = 0.21$, while keeping a high crosslinker concentration $c = 10\,\%$. Each microgel is composed of $N_{\mathrm{m}} \approx 13000$, and we simulate $N = 54$ microgels for different values of the packing fractions, similarly to what was reported for $\delta_{\mathrm{rel}} = 0.275$ in the main text.

Figure S6 and Figure S7 report, respectively, results for $g(r)$ and for the shape phase diagram for these microgels at different packing fractions. For both observables, we find a qualitatively similar behavior to the $\delta_{\mathrm{rel}} = 0.275$ case examined in the manuscript, providing robustness and generality to our findings. The latter example has been preferred since it is directly comparable to experimental data of Ref.[2]

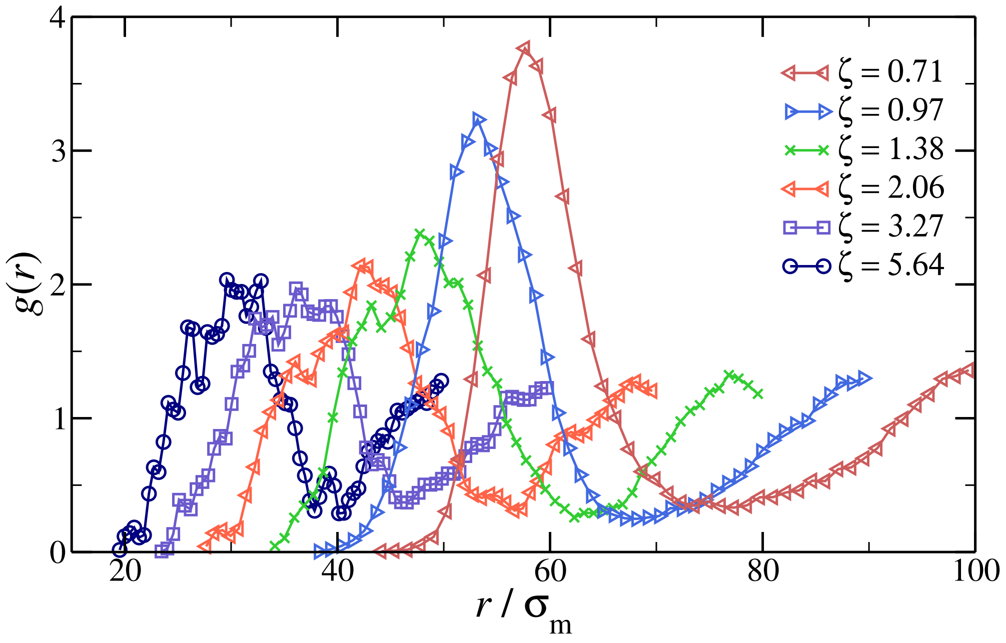

Figure S6: Radial density distribution functions $g(r)$ at different packing fractions ζ of hollow microgels with $c = 10\,\%$ and a thinner shell $\delta_{\text{rel}} = 0.21$, with respect to that reported in the main text.

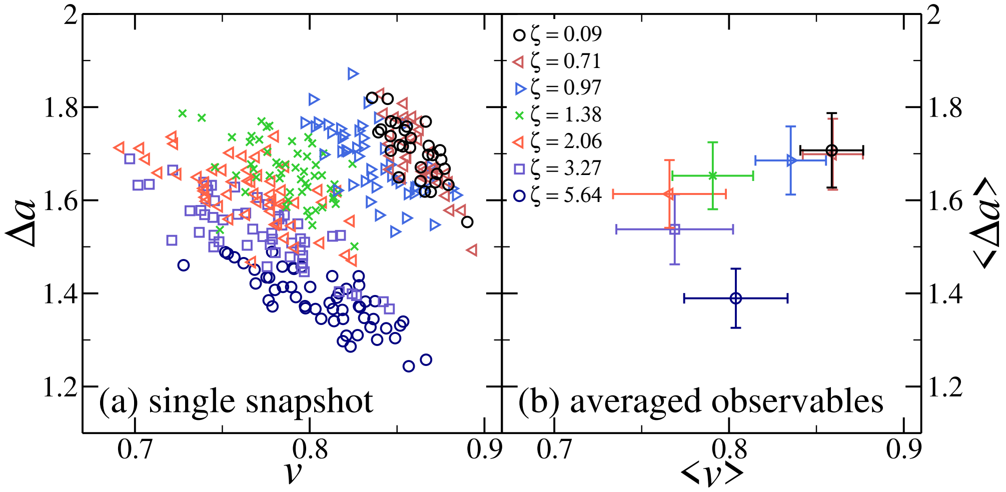

Figure S7: Shape phase diagrams of $c = 10\,\%$ and $\delta_{\text{rel}} = 0.21$ hollow microgels. On the left, the phase diagram at a single point in time is plotted, while the right plot shows an ensemble and time average.

S4: Choosing the optimal parameters for calculating the microgel surface mesh

We now examine the effect of the parameters used to calculate the surface mesh via OVITO.[3,4] In particular, there are two tunable input values defining the output mesh: the probing radius r_{P} and the smoothing level s. While the former, referring to the radius of the probing sphere the software uses to create the mesh, dictates the resolution of the mesh and how tightly it will lie on our microgel, the latter is an integer number controlling how many times the inbuilt smoothing procedure of the mesh is applied. It is therefore important to decide which parameter combination is appropriate to use, so that we are able to capture the microgels' overall shape as accurately as possible. In addition, we aim to fix these values for each type of microgels for all packing fractions, thus addressing different ranges of deformation. This ensures that we compare shape descriptors that have been computed under identical conditions, thereby enabling a consistent and meaningful comparison across packing fractions.

To determine parameter settings for each simulation, we exemplarily plot the shape phase diagrams of two different simulations at different packing fractions that differ in microgel species in Figures S8 and S9 using several values of r_{P} and s.

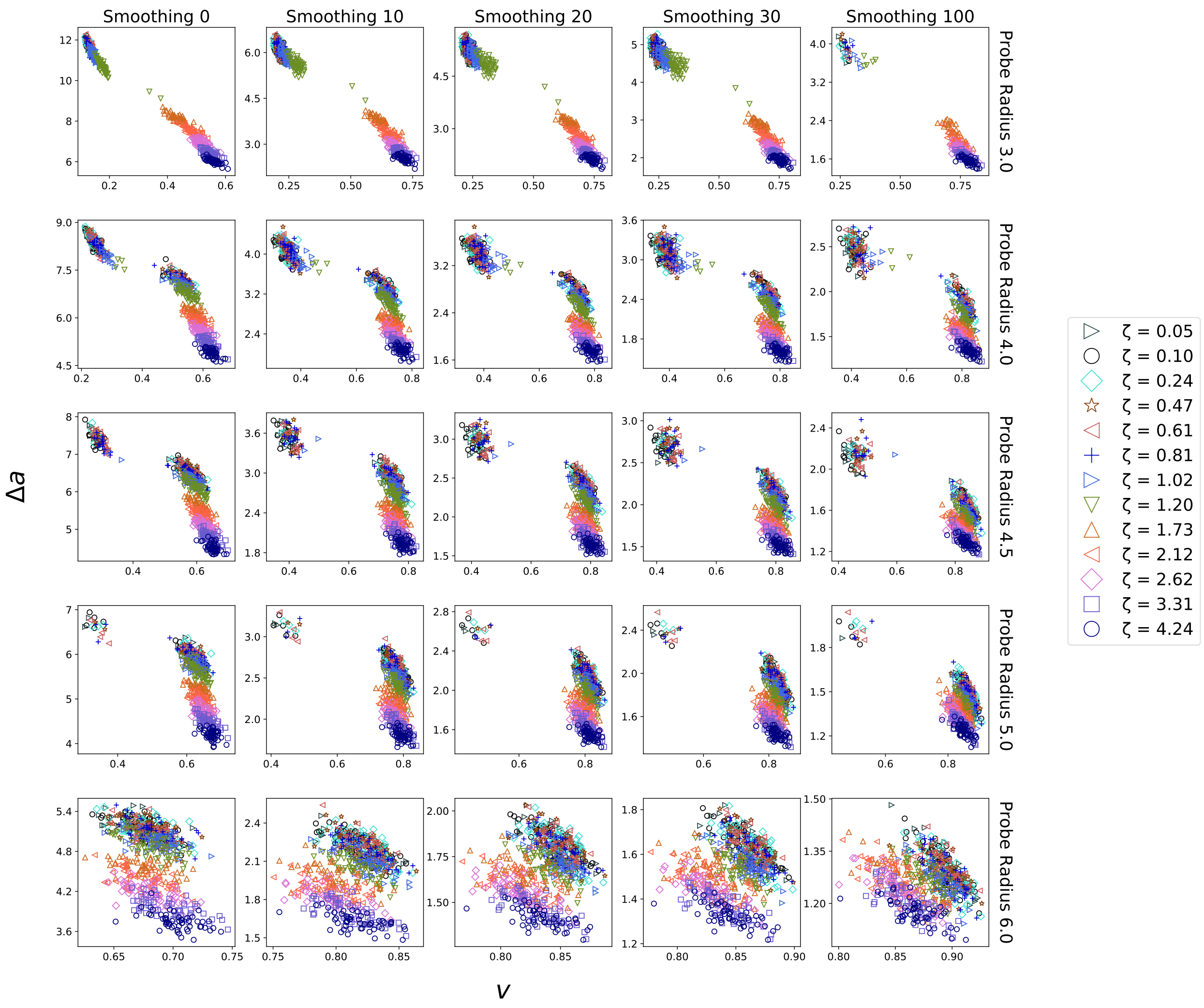

Figure S8: Shape phase diagrams of $c = 5\,\%$ hollow microgels at different packing fractions ζ using various parameter combinations (probe radii in units of σ_{m} and smoothing settings) to construct the surface mesh. Each dot represents one individual microgel at a given timestep t.

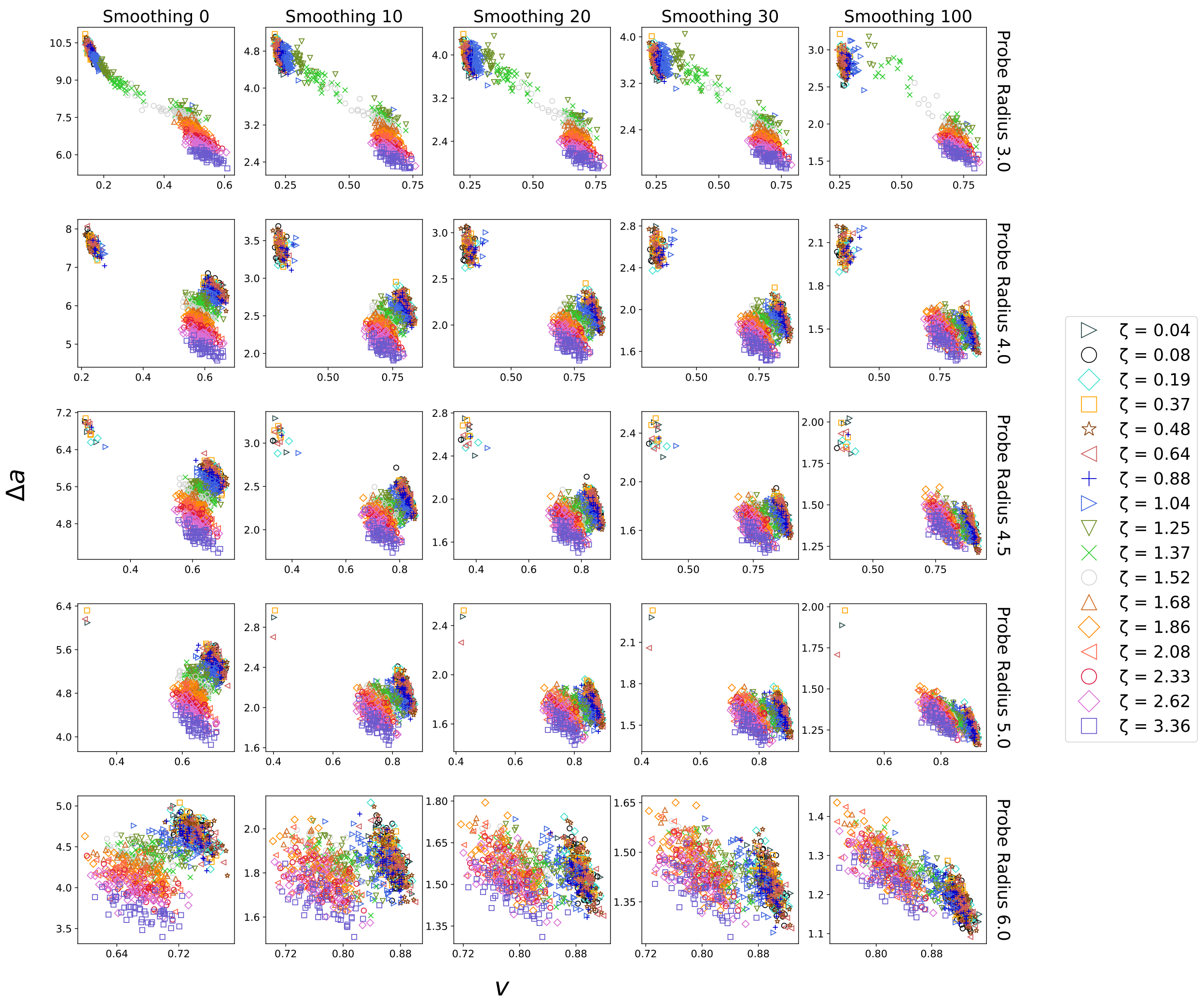

Figure S9: Same as Fig. S9 for $c = 10\,\%$ hollow microgels.

The similar behaviour of all panels confirms that the underlying physics remains visible independently of the parameter choice. Still, especially in Figure S8, we notice that low values of r_{P} cause problems for a large portion of the microgels analysed: in the dilute regimes (packing fractions up to $\zeta \sim 1$), many microgels are shifted up and to the left. For our analysis, we only consider the microgel shape from the outside. Hence, we only use the region of the surface mesh that wraps around the whole object, disregarding the vertices created along the microgel's cavity, which is still present at low packing fractions. But sometimes there exists only one distinct surface mesh region if we have a very tight fit, as shown in Figure S10a. Instead, the surface mesh in Figure S10b displays two different regions,

where we are able to select the one outside the microgel to draw appropriate conclusions on the overall microgel shape. The hole in Figure S10a causes the reduced volume v to shrink by excluding the cavity volume from V and including the surface area along the cavity to get A - see equations 8 and 9 in the paper. Simultaneously, Δa reaches values that are not meaningful in our context due to the high mean curvature H.

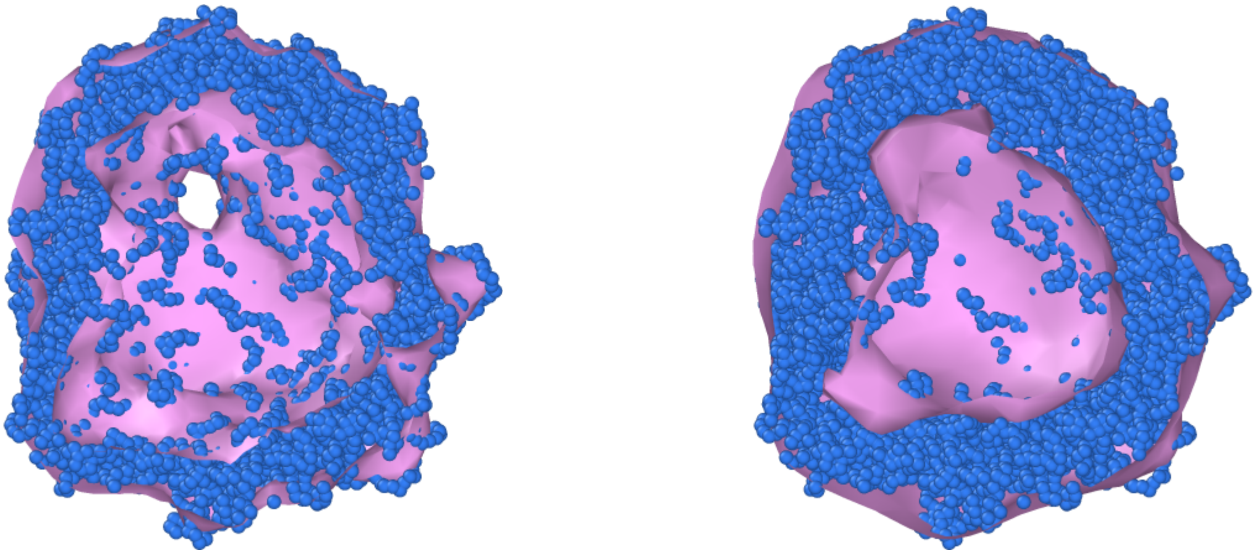

(a) (b)

Figure S10: Example of surface mesh resolution choice: Sliced snapshot capturing simulations of a representative hollow microgel at low ζ using (a) probing radius $r_{\mathrm{P}} = 5.0\,\sigma_{\mathrm{m}}$ and smoothing value 30, and (b) probing radius $r_{\mathrm{P}} = 3.0\,\sigma_{\mathrm{m}}$ and $s = 30$. The low values of r_{P} cause the surface mesh to no longer consist of two distinct regions (along the outside and around its cavity).

For the smoothing level, it is preferable to use high values of s to focus on the general microgel shape without considering its bumpy surface too much to properly classify the different shapes in the bulk. On the other hand, too high values of the smoothing might cause singularities in the surface mesh and therefore lead to unreasonably high curvatures and Δa. In the extreme case of $s = 100$, Δa becomes even larger than 50 for some microgels - not shown in the plots for visual purposes. Hence, a good compromise is to use a high enough smoothing level ($s = 30$) while avoiding singularities, as well as a possible washing out of the microgel shape.

However, despite careful parameter choices, there still exist some individual microgels that take unreasonable values of reduced volume and Δa due to aforementioned reasons, so that we have to exclude these unphysical points in our plots. Our goal is simply to minimise the number of outliers with appropriate values of r_{P} and s, specified in the main text for both sets of microgels, in order to provide a meaningful data set.

References

(1) Del Monte, G.; Zaccarelli, E. Numerical study of neutral and charged microgel suspensions: From single-particle to collective behavior. *Physical Review X* **2024**, *14*, 041067.

(2) Hazra, N.; Lammertz, J.; Babenyshev, A.; Erkes, R.; Hagemans, F.; Misra, C.; Richtering, W.; Crassous, J. J. Charged hollow microgel capsules. *Soft Matter* **2024**, *20*, 4608–4620.

(3) Stukowski, A. Visualization and analysis of atomistic simulation data with OVITO–the Open Visualization Tool. *Modelling and Simulation in Materials Science and Engineering* **2009**, *18*, 015012.

(4) Stukowski, A. Computational Analysis Methods in Atomistic Modeling of Crystals. *JOM* **2014**, *66*, 399–407.
